# The London Quarterly Journal of Dental Science

**Published:** 1859-04

**Authors:** 


*The London Quarterly Journal of Dental Science.*—We have been informed that this publication has been discontinued, but hope our information is incorrect. We can scarcely believe that our transatlantic brethren would permit as valuable a work as this to stop for want of support. We know not by whom it was conducted, but it was certainly edited with ability, and deserved to be well sustained. Every dentist in Great Britain should have taken it, and it ought to have had, at least two hundred subscribers in this country. We trust our information with regard to the discontinuance of the work, is incorrect.

There is, or was, another dental periodical published in London—the "British Journal of Dental Science," which we have not received for more than six months. What has become of it? We hope it also has not been discontinued.

The advent of these journals was hailed by us as the dawning of a new era on dentistry in England. The loss of two such able co-laborers in this comparatively new field of science, would be a source of profound regret. We are unwilling to believe that the members of the profession on the other side of the Atlantic have permitted either of these publications to cease.

Since the above was in type we have learned that the Quarterly has been merged into a Monthly.

